# Evaluation of targeted next-generation sequencing for microbiological diagnosis of acute lower respiratory infection

**DOI:** 10.3389/fmicb.2025.1615965

**Published:** 2025-08-18

**Authors:** Fengzhen Yang, Lihua Jiang, Qingmei Cao, Maoli Yi, Qi Zhao

**Affiliations:** Department of Laboratory Medicine, Qingdao University Affiliated Yantai Yuhuangding Hospital, Yantai, China

**Keywords:** next-generation sequencing, acute lower respiratory infection, microorganism, resistance gene, microbiological diagnosis

## Abstract

**Purpose:**

To evaluate the performance of targeted next-generation sequencing (tNGS) in pathogen detection in acute lower respiratory infection.

**Methods:**

The retrospective study was conducted between July 2023 and May 2024 at the Yantai Yuhuangding Hospital. Patients with acute lower respiratory infections were included. Qualified sputum or bronchoalveolar lavage fluid samples were collected for tNGS and conventional microbiological tests(CMTs), including culture, staining, polymerase chain reaction (PCR), and reverse transcription-PCR (RT-PCR). The time required and cost were counted.

**Results:**

A total of 968 patients were enrolled. Study analysis discovered 1,019 strains of bacteria, 259 strains of fungi, 302 strains of viruses, 76 strains of *Mycoplasma pneumoniae*, and two strains of *Chlamydia psittaci* using tNGS. In addition, tNGS also identified 39 *mecA*, four *KPC*, 19 *NDM*, and two *OXA-48* genes. The positive rates for bacteria, fungi, viruses, mycoplasma, and chlamydia obtained using tNGS were significantly higher than those determined using traditional methods. Among them, tNGS showed high consistence with mycobacterium DNA test, *influenza A (H1N1) virus* nucleic acid test and *COVID-19* nucleic acid test. Poor consistency between drug resistance genes and bacterial resistance phenotypes was found. In addition, tNGS also had advantages over traditional methods in terms of detection time and cost.

**Conclusion:**

Compared to traditional methods, tNGS had higher sensitivity in detecting bacteria, fungi, viruses, and other pathogens in acute lower respiratory infection, and also had the advantages of timeliness and cost-effectiveness, making it a promising method for guiding clinical diagnosis.

## Background

Infectious diseases cause over 17 million deaths annually, accounting for over 25% of total mortality in the world (Dai et al., 2022). Among these infectious diseases, acute lower respiratory infection, especially pneumonia, is still one of the main causes of infection-related deaths (Thorburn et al., 2015; Yang et al., 2018). Rapid and accurate detection of pathogenic microorganisms is a prerequisite for accurate diagnosis of acute lower respiratory infection and a key to determining treatment strategies (Wu et al., 2023). A failure to make a timely diagnosis in patients with respiratory infection contributes to poor outcomes. However, traditional methods for microbial identification and diagnosis of infections, represented by culture, are often time-consuming and have low sensitivity (Mansoor et al., 2023; Kullar et al., 2023; Li et al., 2023). Moreover, several types of microorganisms, such as anaerobic bacteria, viruses, and *Mycoplasma pneumoniae* are very difficult to cultivate (Gao et al., 2021; Chen et al., 2024; Patiño et al., 2021; Huang et al., 2023). Therefore, traditional culture-based approaches cannot meet the requirements for clinical diagnosis of pathogenic infections in terms of accuracy and timeliness. Metagenomic NGS (mNGS) is an increasingly rapid and high-throughput method for pathogen detection, which also has the ability to detect unknown pathogens (Gao et al., 2021; Chen et al., 2024). However, it is expensive and imposes a significant economic burden on patients. The conventional PCR/RT-PCR has high sensitivity, but it can only detect specific pathogens each time, which limits the clinical application. Based on these, it is crucial to develop a fast, cost-effective detection method that covers a wide range of pathogens.

Targeted next-generation sequencing (tNGS) enriching specific pathogen sequences and antimicrobial resistance markers has become an alternative option to circumvent these limitations. Although tNGS has certain limitations, firstly, it cannot differentiate between colonization and infection of pathogens. Secondly, the genetic material of dead pathogens can result in false positives using this method. However, its detection speed is getting faster and the cost is relatively low, and it does not rely on traditional culture (Yu et al., 2023; Alexis Trecourt et al., 2023; Li et al., 2021; Huang et al., 2023). Furthermore, it can simultaneously detect pathogens and their drug-resistant genes (Zhang et al., 2024; Song et al., 2022; Iyer et al., 2023). Given these advantages, tNGS has significant application prospects in the diagnosis of acute lower respiratory infection. Previous research on tNGS has mostly focused on the diagnosis of tuberculosis and meningitis (Mansoor et al., 2023; Li et al., 2023; Gao et al., 2021; Chen et al., 2024), and relatively few studies have evaluated the performance of tNGS in the context of acute lower respiratory infection.

The present study aimed to evaluate the performance of tNGS in pathogen detection in acute lower respiratory infection by comparing its detection rate to those of conventional microbiological tests(CMTs), including culture, staining, polymerase chain reaction (PCR), and reverse transcription-PCR (RT-PCR).

## Methods

### Study population

The present study was conducted between July 2023 and May 2024 at the Yantai Yuhuangding Hospital of Shandong Province, a 3,000-bed tertiary teaching hospital located in East China. Patients with obvious symptoms of acute lower respiratory infection were considered for inclusion in the study. The cohort included the following: (I) pneumonia patients with any of the following symptoms or signs: fever (>38 °C), tachypnea, tachycardia, wheezing, cough, new or progressive exudation, solid shadow, and cavity or pleural effusion on chest images; (II) tracheitis or tracheobronchitis patients with two of the following symptoms or signs: cough accompanied by increased sputum, respiratory distress, wheezing, apnea, or tachycardia; and (III) patients with other infections of the lower respiratory tract based on pulmonary radiology results, such as lung abscess or empyema. Specimens repeatedly submitted by the same patient within 1 week were excluded. The procedures involving human subjects were in accordance with the Declaration of Helsinki (as revised in 2013). Qualified sputum or bronchoalveolar lavage fluid (BALF) samples from all patients were collected for culture, rapid acid-fast staining, gomori methenamine silver(GMS) staining, PCR or RT-PCR, and tNGS. The time required and cost were also counted.

### Clinical data collection

Clinical and demographic data for all study participants were retrieved through medical records and included information on sex, age, underlying diseases, admission to department, length of hospitalization, mechanical ventilation, and outcomes.

### Conventional microbiological tests

All specimens were inoculated on Columbia blood agar (Autobio Diagnostics Co., Ltd., Zhengzhou, China), MacConkey agar (Autobio), chocolate agar(Autobio), and Sabouraud agar (Autobio) plates for bacterial and fungal cultivation. The microbiological identification was performed using matrix-assisted laser desorption/ionization time-of-flight mass spectrometry (MALDI-TOF MS, Bruker Daltonics, Karlsruhe, Germany). Antibiotic susceptibility tests (imipenem and meropenem for Gram negative bacteria, and oxacillin for *Staphylococcus aureus*) were carried out via the VITEK®2 compact system (Biomérieux, Marcy l’Etoile, France). Rapid acid-fast staining (Baso Biotechnology Co., Ltd., Zhuhai, China) and mycobacterial DNA examination(Capital Biotechnology Co., Ltd., Beijing, China) were used to detect mycobacteria. GMS staining (Baso) was performed on patients with a suspected *Pneumocystis jiroveci* infection. PCR or RT-PCR examination (Sansure Biotechnology Co., Ltd., Changsha, China) was used to identify viral nucleic acids in specimens suspected of a viral infection. Immunochromatography (Dynamiker Biotechnology Co., Ltd., Tianjin, China) was utilized to detect carbapenem enzyme.

### The tNGS

The panel design was derived from expert consensus and literature in the field of infection (Huang et al., 2020; Li et al., 2021; Liu et al., 2023; Kasper and Fauci, 2016). The tNGS panel covered 153 pathogen targets commonly encountered in clinical scenarios and some resistance genes, and the complete list of target species and resistance genes identified is shown in Supplementary Table 1. The reference sequence data was curated mainly from NCBI RefSeq/NT, and highly similar redundant sequences were removed for improvement. For target selection, priority was given to genes that had been verified by PCR methods, followed by bioinformatics evaluation of conserved and specific regions. Specific primers were designed in accordance with strict standards described in a previous study, and the full list of primer panels can be found in Supplementary Table 2 (Yin et al., 2024).

The tNGS workflow includes total nucleic acid extraction, library construction, sequencing, and bioinformatics processing. Total nucleic acid was extracted from all samples, including clinical samples, negative controls (NC) and positive controls (PC), using the Nucleic Acid Extraction and Purification Kit (KS118, KingCreate Biotech, Guangzhou, China) on the KingFisher™ Flex Purification System (Thermo Fisher Scientific, Waltham, MA, United States). PCR amplification was performed using the Respiratory Pathogen Microorganisms Multiplex Testing Kit (KS608-100HXD96, KingCreate, Guangzhou, China). The amplification protocol started with an initial denaturation at 95 °C for 3 min, followed by 25 cycles of DNA denaturation at 95 °C for 30 s and annealing at 68 °C for 1 min. Subsequently, the samples underwent 30 consecutive heating cycles, including denaturation at 95 °C for 30 s, annealing at 60 °C for 30 s, and extension at 72 °C for 30 s. Finally, an extension was performed at 72 °C for 1 min to ensure the completion of all partially amplified fragments. After PCR amplification, the resulting product was purified. The generated library was then quantified using the Invitrogen™ Qubit™ 3.0/4.0 Fluorometer (Q33216, Thermo Fisher Scientific, USA) to ensure that the library concentration of all samples was ≥ 0.5 ng/μL; otherwise, library reconstruction was carried out.

Sequencing was performed using the KM Miniseq Dx-CN Sequencer (KY301, KingCreate, Guangzhou, China). Fastp v0.20.1 was employed for adapter trimming and low-quality read filtering. The read filtration criteria were delineated as follows: (1) reads with an average quality score below 15 were subjected to trimming; (2) reads with a length of < 15 bp; (3) reads possessing the ambiguous “N” bases over 10. The obtained sequences were aligned with the human genome to filter out host sequences. Subsequently, Bowtie2 v2.4.1 was used to align with the reference database (containing 683 species of bacteria, 372 species of viruses, and 349 species of fungi) in a “very-sensitive” mode. The number of reads per 100,000 sequencing reads was calculated at the species and genus levels.

### Statistical analyses

SPSS Statistics 26 software (IBM Corporation, NY, United States) was used for data entry. Categorical data were summarized using percentages. For comparison of categorical variables, the chi-square test or Fisher’s exact test was performed. Continuous variables were represented as means ± standard deviations and compared using the Student’s *t*-test or Mann–Whitney U-test as appropriate. *p* < 0.05 was considered statistically significant.

## Results

### Baseline patient characteristics

A total of 968 patients were enrolled based on the inclusion criteria, of which 54.9% were male and 45.1% were female. Patient age ranged from 1 to 98 years, with an average of 60.72 ± 20.06 years. Furthermore, 6.3% of patients came from pediatric wards, 55.5% from respiratory wards, 34.5% from intensive care units, and 3.7% from other departments. 52.5% of patients had other comorbidities, with chronic obstructive pulmonary disease, bronchiectasis, and lung cancer being the most common. The average hospitalization time was 17.04 ± 24.54 days. In addition, 73.1% of patients improved and were discharged, 16.8% died, 0.5% were transferred to higher-level hospitals, and 9.5% were transferred to infectious disease hospitals. The average white blood cell (WBC) counts, C-reactive protein (CRP) levels, and procalcitonin (PCT) levels were 9.47 ± 5.79, 60.40 ± 69.97, and 1.62 ± 6.53, respectively.

Among the 968 evaluated patients, 18.1% underwent mechanical ventilation, while 81.9% did not. The comparison between the two groups is shown in Table 1. Compared to the non mechanical ventilation group, the mechanical ventilation group had a higher age, higher WBC counts, CRP and PCT levels, longer hospital stay, and higher mortality rate.

**Table 1:** Baseline characteristics between mechanical ventilation group and non mechanical ventilation group.

Variates	Mechanical ventilation group (*n* = 175)	Non mechanical ventilation (*n* = 793)	*p* value
Age (years)	70.68 ± 14.41	58.52 ± 20.49	0.000
Sex (male)	93 (53.14%)	438 (55.23%)	0.615
WBC (*109/L)	11.89 ± 8.16	8.92 ± 4.94	0.000
CRP (mg/L)	93.45 ± 76.06	53.52 ± 66.72	0.003
PCT (ng/mL)	4.03 ± 11.02	0.92 ± 4.22	0.000
Hospital stay (days)	37.87 ± 40.74	12.49 ± 16.04	0.000
Outcome (died)	107 (61.14%)	56 (7.06%)	0.000

A total of 305 qualified sputum samples and 663 BALF samples were collected in the study, and there was no statistically difference in the above indicators among different specimen types.

### Overview of tNGS and CMTs

**Figure 1:**
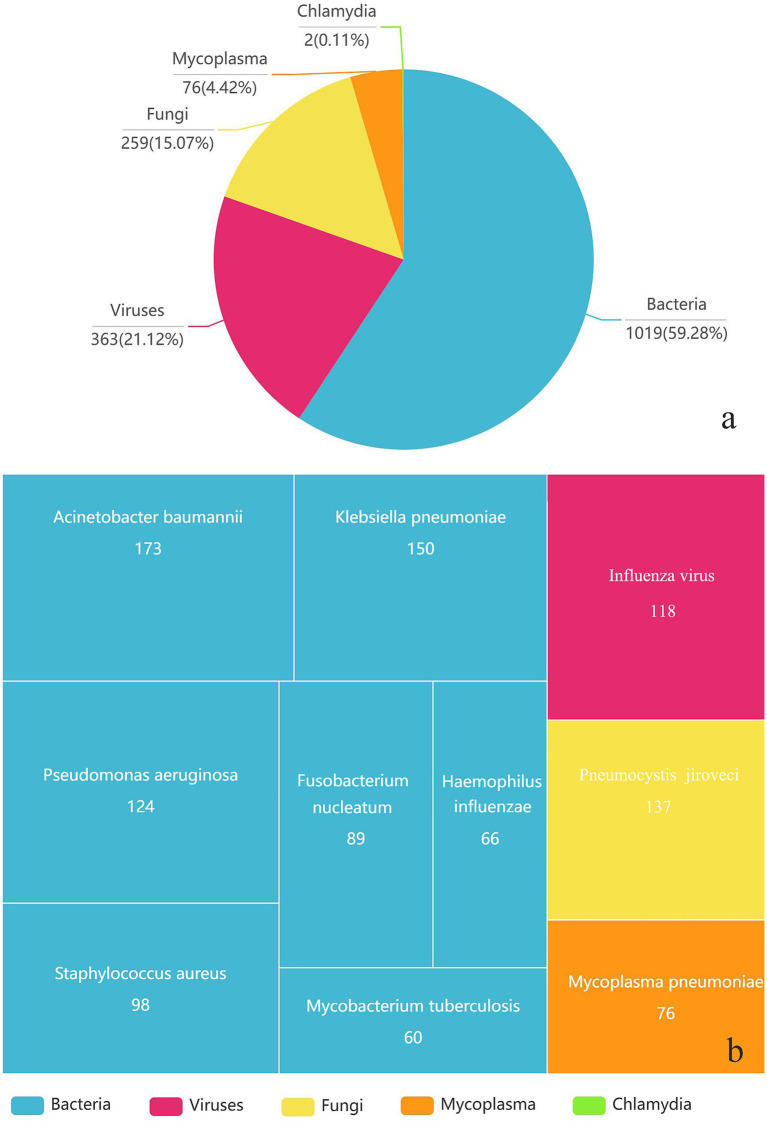
The types of pathogens detected **(a)** and the distribution of the top 10 pathogens **(b)** in 968 respiratory samples using tNGS.

**Figure 2:**
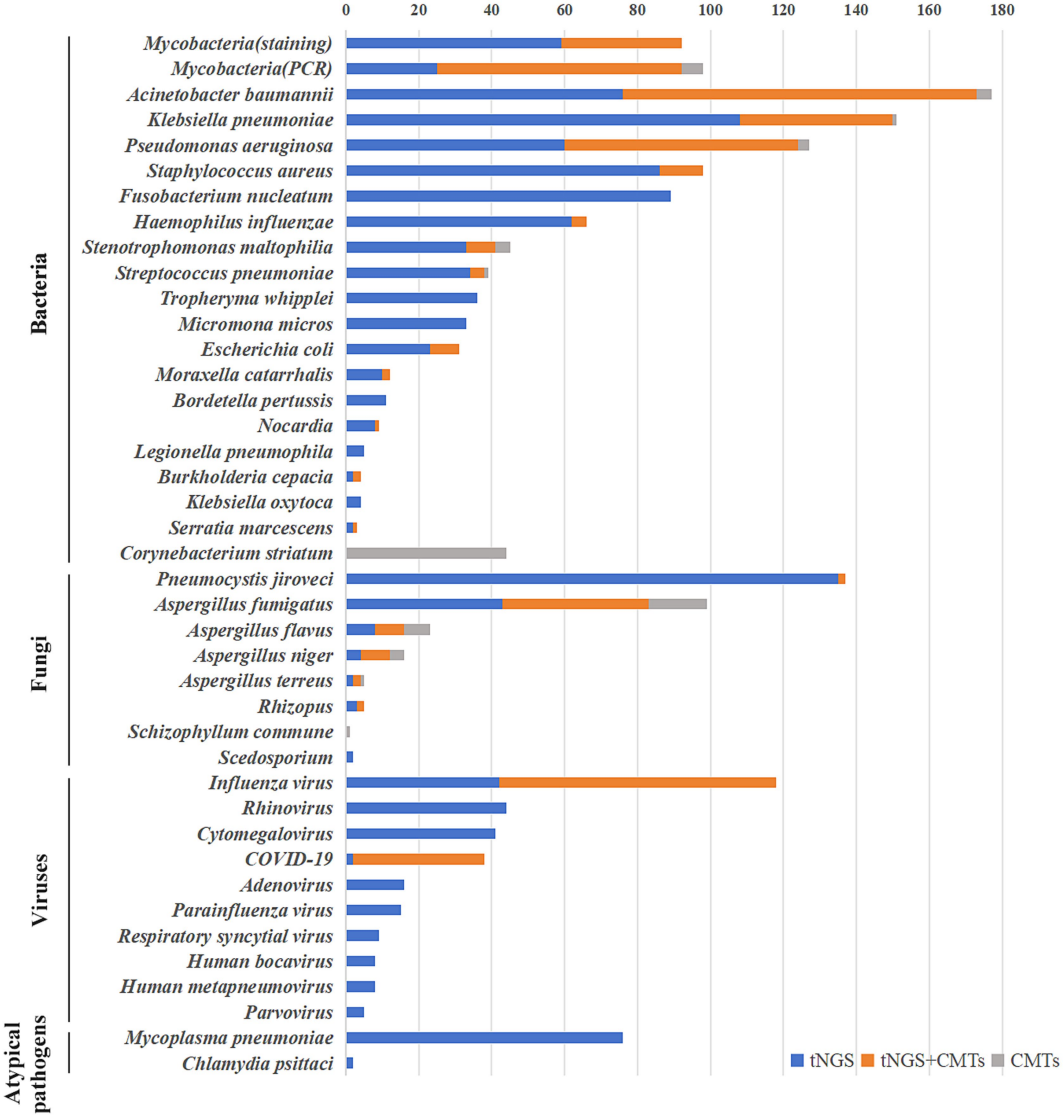
The comparison of pathogens between tNGS and CMTs.

Overall, 1,019 strains of bacteria, 259 strains of fungi, 302 strains of viruses, 76 strains of *Mycoplasma pneumoniae*, and two strains of *Chlamydia psittaci* were detected in 968 samples through tNGS. Pathogen distribution and the top 10 pathogens are shown in Figure 1. Only one pathogen (92 were bacteria, 28 were fungi, 60 were viruses, and 27 were mycoplasma) was detected in 207 cases (21 sputum, 186 BALF), while 761 cases were characterized by at least two pathogens. BALF was more likely to detect one pathogen (28.05% vs. 6.88%), and the difference is statistically significant. Resistance genetic testing identified 39 *mecA*, four *KPC*, 19 *NDM*, and two *OXA-48* genes. The comparison of pathogens between tNGS and CMTs is shown in Figure 2.

### Bacteria detection

A total of 92 cases of mycobacteria were detected in 968 samples, including 60 cases of *Mycobacterium tuberculosis* and 32 cases of *non-tuberculous mycobacteria* (*intracellular mycobacteria*, accounting for 78% of *non-tuberculous mycobacteria*, followed by *Mycobacterium abscessus* and *Mycobacterium avium*). The results of tNGS and rapid acid-fast staining as well as mycobacterial DNA detection(PCR) are shown in Figure 2. Compared to rapid acid-fast staining and PCR, tNGS could increase the mycobacterial detection rate by 178.8 and 26.0%, respectively, and the detection rate of Mycobacterium by tNGS was significantly higher than rapid acid-fast staining method (*p =* 0.000). The kappa coefficients for the consistency of tNGS with rapid acid-fast staining and PCR were 0.503 and 0.795, respectively. Using PCR as the gold standard, the sensitivity and specificity of tNGS for detecting Mycobacterium were 91.8 and 97.2%, respectively.

As shown in Figure 2, compared to traditional cultivation methods, tNGS has significantly improved the detection of bacteria. Particularly for bacteria that are not easily detected by conventional cultivation, such as *Fusobacterium nucleatum*, *Tropheryma whipplei* and *Micromona micros*, tNGS detected 89, 36, and 33 cases respectively, whereas the cultivation methods failed to detect any. For the majority of bacteria, those detected by culture methods were also identified by tNGS. However, there were 44 cases of *Corynebacterium striatum*, four cases of *Acinetobacter baumannii*, three cases of *Pseudomonas aeruginosa*, one case of *Klebsiella pneumoniae*, one case of *Streptococcus pneumoniae*, and four cases of *Stenotrophomonas maltophilia* with a positive culture but negative tNGS result. In terms of bacterial detection, tNGS and culture only showed 40.6% full or partial consistency. Using culture as the gold standard, the sensitivity and specificity of tNGS for detecting bacteria were 81.1 and 22.2%, respectively.

### Fungal detection

A total of 122 cases of filamentous fungi were detected using tNGS, with *Aspergillus fumigatus* being the most common, detected in 83 cases, followed by *Aspergillus flavus* and *Aspergillus niger*, detected in 16 and 12 cases, respectively. A total of 89 filamentous fungi were identified using the cultivation method, and the fungal distribution patterns were similar to those observed by tNGS. *Aspergillus fumigatus*, *Aspergillus flavus*, and *Aspergillus niger* were detected in 56, 15, and 12 cases, respectively. In addition, one case of *Schizophyllum commune* was detected by the cultivation method but not by tNGS, and two cases of *Scedosporium* were detected by tNGS but not by the cultivation method. Moreover, 137 cases of *Pneumocystis jiroveci* were detected by tNGS, while only two cases were detected by GMS staining. The positive rates of tNGS for filamentous fungi and *Pneumocystis jiroveci* were significantly higher than those of traditional methods (*p* < 0.05). The kappa coefficient of consistency between tNGS and culture methods for detecting filamentous fungi was 0.495. Using culture as the gold standard, the sensitivity and specificity for detecting filamentous fungi were 67.4 and 92.9%, respectively.

### Virus, mycoplasma, and chlamydia infection detections

Three hundred and two strains of viruses were detected in 968 patients, as shown in Figure 2. *Influenza virus* was the most common viruses, with 118 cases detected, followed by *Rhinovirus*, *Cytomegalovirus* and *COVID-19*, with 44, 41 and 38 cases detected, respectively. Among the 118 cases of *influenza virus*, 80 were *influenza A* (*H1N1*) *virus* and 38 were other *influenza viruses*. Unfortunately, nucleic acid tests were carried out only for *influenza A (H1N1) virus* and *COVID-19*, and 76 cases and 36 cases were detected, respectively. The kappa coefficients between tNGS and nucleic acid test for *Influenza virus* and *COVID-19* were 0.761 and 0.972, respectively. Using nucleic acid testing as the gold standard, the sensitivity and specificity of tNGS for detecting *influenza A (H1N1) virus* were 97.4 and 99.3%, respectively. The sensitivity and specificity for detecting *COVID-19* were 97.2 and 99.7%, respectively.

In addition, a total of 76 cases of *Mycoplasma pneumoniae* and two cases of *Chlamydia psittaci* were detected in tNGS. The positive rate of *Mycoplasma pneumoniae* in pediatric patients was much higher than that in patients from other departments [59.02% (36/61) vs. 4.41% (40/907)].

### Resistance gene detection

**Figure 3:**
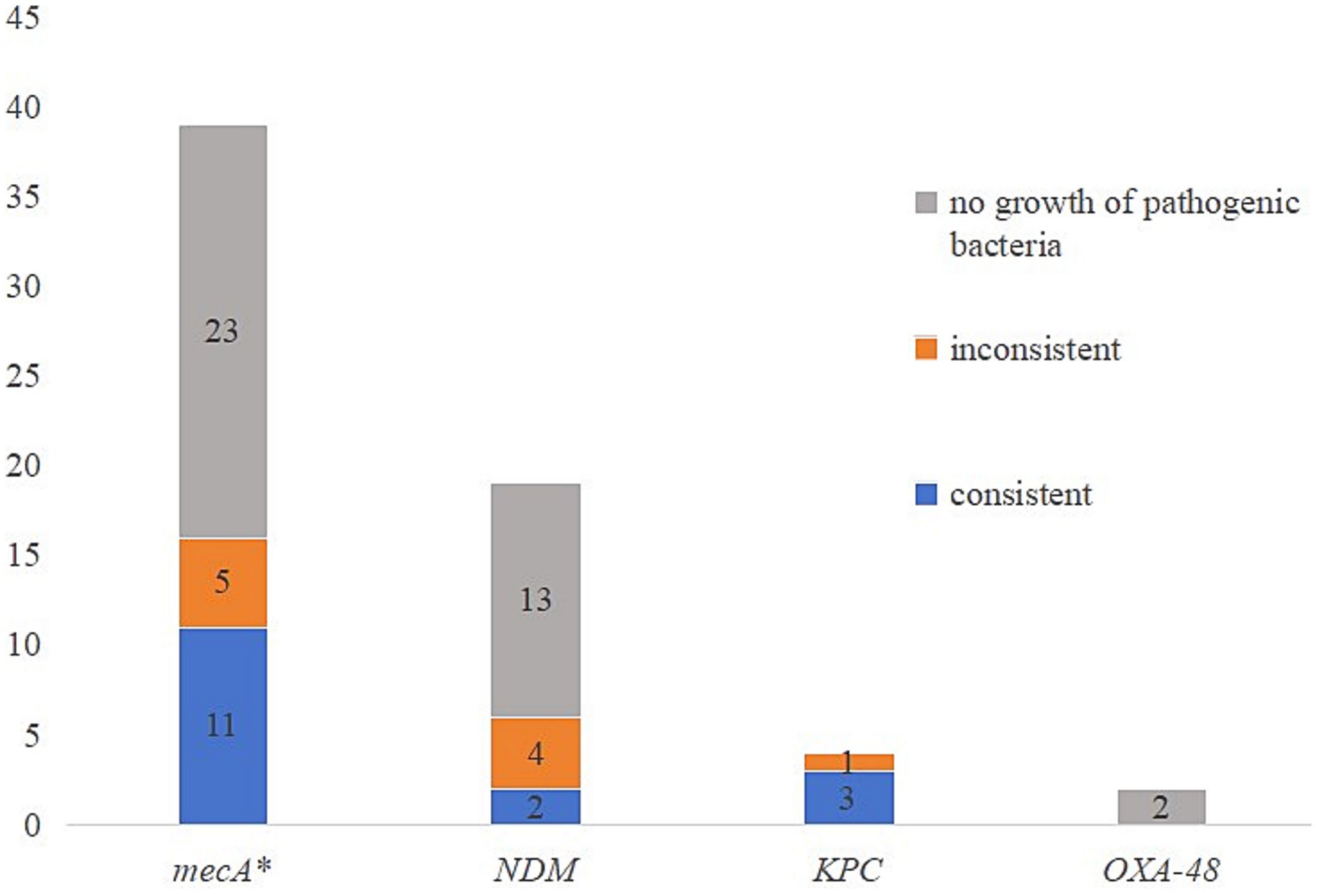
The comparison of resistance genes and resistance phenotypes. *One case of *Staphylococcus aureus* was resistant to oxacillin, while *mecA* was negative.

Multiple resistance genes or genotypes can also be detected through tNGS, mainly including resistance genes that pose a serious threat to patients. The results are shown in Figure 3, the comparison between resistance genes and resistance phenotypes revealed that the overall consistency rate between resistance genes and resistance phenotypes was only 25% (16/64). Except for the high consistency rate (75%) between *KPC* resistance genes and resistance phenotypes, the consistency rate between other resistance genes and resistance phenotypes was less than 30%. In addition, a case of *Staphylococcus aureus* was found to be resistant to oxacillin, while *mecA* genetic test was negative.

### Time required and cost

It took approximately 24 and 72 h for pathogen diagnosis by tNGS and culture, respectively. In addition, tNGS could simultaneously detect 153 pathogens and 370 resistance genotypes at a cost of approximately 1,000 RMB, making it cost-effective compared to traditional methods. Therefore, tNGS also had advantages over traditional methods in terms of detection time and cost.

## Discussion

The tNGS is an increasingly fast and relatively low cost method that can screen for multiple human pathogens in sputum and BALF samples in an unbiased manner (Dai et al., 2022; Li et al., 2022; David et al., 2022). One hundred and fifty-three pathogens and over 370 resistance genes or genotypes can be detected for only about 1,000 RMB, and the results can be obtained within 24 h, faster than culture, all of which lead to the increasing application of tNGS. The present study evaluated the application of tNGS in acute lower respiratory infection and found that the pathogen distribution detected by tNGS was consistent with the recognized pathogen distribution in acute lower respiratory tract infection, with bacterial and viral infections being the most common. Of course, previous studies have shown that some pathogens, such as *Streptococcus pneumoniae*, may also be normal microbial communities that colonize the respiratory tract (Li et al., 2022; Li et al., 2021). These pathogens may contaminate sputum when it passes through the mouth, leading to positive results. Therefore, characterizing the symbiotic colonization or pathogenic infection of these microorganisms requires consideration of the patient’s clinical manifestations, inflammatory indicators, and imaging results.

The tNGS test for mycobacterium was significantly superior to rapid acid-fast staining. The ability of tNGS to detect mycobacterium was comparable to that of PCR, and with high consistency. The sensitivity and specificity of tNGS for detecting Mycobacterium were both greater than 90%, indicating that tNGS had a stronger ability to detect Mycobacterium. Meanwhile, high sensitivity of tNGS made its detection rate for bacteria significantly higher than that of the culture methods. In this study, the consistency between tNGS and culture was only 40.6%, and the specificity was poor because tNGS could not distinguish between colonization and infection, live pathogens and corpses, and a small amount of pathogen colonization or genetic material could make tNGS positive, which is consistent with previous studies (Deng et al., 2023; Zhang et al., 2024). Moreover, tNGS cannot provide antimicrobial susceptibility results, therefore tNGS cannot replace culture in bacterial detection. Nevertheless, there are many bacteria that are difficult to cultivate, such as *Legionella pneumophila*, *Bordetella pertussis*, and *Tropheryma whipplei*, as well as some anaerobic bacteria, such as *Micromona micros* and *Fusobacterium nucleatum*. These bacteria are picky pathogens that are difficult to detect using traditional cultivation methods, while tNGS detection is not affected and can improve its detection rate. Unfortunately, the bacteria identified in the present study did not include *Corynebacterium striatum*, although prior studies generally consider it to be colonizing bacteria. It has also been recently reported that *Corynebacterium striatum* caused lower respiratory infections (Shariff et al., 2018; Zhang et al., 2023).

The detection rate of tNGS for filamentous fungi was also higher than that of culture, but the matching degree was not particularly satisfactory. Due to the thick fungal cell walls, it was difficult to extract DNA, which may lead to negative tNGS and positive culture method results. Moreover, the culture methods were greatly influenced by antibiotics, resulting in positive results for tNGS and negative results for the culture methods. In addition, several samples showed inconsistent results using culture and tNGS, which may be due to double infections or high homology between the two fungi, resulting in incorrect identification by tNGS or MALDI-TOF MS. Although the sensitivity and consistency of tNGS were not particularly ideal, the specificity was greater than 90% and can be used as an auxiliary diagnostic method for filamentous fungal infections. *Pneumocystis jiroveci* does not grow in commonly used culture media, and traditional methods often use GMS staining for detection. The present study found that the positivity rate of GMS staining was very low, while tNGS significantly increased the positivity rate.

The tNGS had excellent advantages in detecting viruses and other pathogens, including mycoplasma, chlamydia, and *Rickettsia,* which was also confirmed by the high sensitivity and specificity of tNGS in detecting *influenza A (H1N1) virus* and *COVID-19*. Viruses and mycoplasma are difficult to cultivate, and laboratory testing often uses single PCR or specific antibodies, which is time-consuming and costly (Chen et al., 2022). The tNGS simultaneously detected 43 RNA viruses, 25 DNA viruses, and 11 other pathogens, greatly improving detection efficiency and reducing detection costs. In terms of the viruses, *Influenza virus* and *Rhinovirus* were the top two viruses detected, consistent with the generally recognized pathogens in acute lower respiratory infections. The detection rates of mycoplasma and chlamydia in tNGS were relatively low, which is consistent with previous study results (Li et al., 2022).

The biggest problem with molecular detection of drug resistance genes is the lack of sufficient research data on the consistency between drug resistance genes and resistance phenotypes, which makes it difficult for drug resistance genes to be used as resistance markers in clinical applications. Although research has found that tNGS drug resistance genes were in line with resistance phenotypes in approximately 65% (Zhang et al., 2024), our study found that the consistency between the two was relatively low. The appearance of positive resistance genes with sensitive phenotypes may be related to the unexpressed or low expression levels of resistance genes, and clinical attention should be paid to changes in pathogen resistance. If the resistance gene is positive but the culture method shows no pathogenic bacteria growth, this may be related to the high sensitivity of the tNGS method, which can detect even trace amounts of pathogenic bacteria or genetic material. In addition, since tNGS can detect dead bacteria, if a specimen is collected after the effective use of antibiotics and the pathogen has died, tNGS can still detect its resistance genes, which can also lead to positive resistance genes and negative culture results, rendering the detection of resistance genes meaningless.

This report presents the application of tNGS in acute lower respiratory infection. Nevertheless, some limitations exist in this study. Firstly, this was a single-center study and may not be representative. Secondly, approximately 30% of the specimens in this study were sputum, and the presence of oral colonization bacteria may affect the detection rate, resulting in detection bias. However, it’s undeniable that tNGS surpasses traditional methods in diagnosing acute lower respiratory tract infection in many respects. It has proven to be a promising detection method, guiding the diagnosis of acute lower respiratory tract infection.

## Conclusion

Compared to traditional methods, tNGS had higher sensitivity in detecting bacteria, fungi, viruses, and other pathogens, and also had the advantages of timeliness and cost-effectiveness, all of which are typically considered in clinical use. The tNGS is a promising method for guiding clinical diagnosis of acute lower respiratory infection.

## Data Availability

The raw data supporting the conclusions of this article will be made available by the authors, without undue reservation.
