# Supplementary material for: Evaluation of targeted next-generation sequencing for microbiological diagnosis of acute lower respiratory infection
Source: Front Microbiol. 2025 Aug 18;16:1615965. doi: 10.3389/fmicb.2025.1615965 (PMC12399591; doi:10.3389/fmicb.2025.1615965)
Supplement: Supplementary file 1 [file Table_1.docx]

Supplementary Table 1 the complete list of target species and resistance genes of tNGS

| **RNA viruses(43 types)** | | |
| --- | --- | --- |
| *Influenza A virus* | *Parainfluenza virus type 3* | *Echovirus* |
| *Influenza A(H1N1)pdm09 virus* | *Parainfluenza virus type 4* | *Enterovirus Group C* |
| *Influenza A(H1N1) virus* | *Measles virus* | *Enterovirus Group D* |
| *Influenza A(H3N2) virus* | *Rotavirus* | *Enterovirus Group D68* |
| *Influenza A(H5N1) virus* | *Enterovirus* | *Rhinovirus* |
| *Influenza A(H7N9) virus* | *Coxsackievirus-A2* | *Rhinovirus type A* |
| *Influenza B virus* | *Coxsackievirus-A6* | *Rhinovirus type B* |
| *Influenza C virus* | *Coxsackievirus-A16* | *Rhinovirus type C* |
| *Human metapneumovirus* | *Enterovirus Group 71* | *Respiratory syncytial virus type A* |
| *Coronavirus 229E* | *Enterovirus Group B* | *Respiratory syncytial virus type B* |
| *Coronavirus HKU1* | *Coxsackievirus-A9* | *Mumps orthorubulavirus* |
| *Coronavirus NL63* | *Coxsackievirus-B2* | *Rubella virus* |
| *Coronavirus OC43* | *Coxsackievirus-B3* | *Enterovirus Group A* |
| *Parainfluenza virus type 1* | *Coxsackievirus-B5* |  |
| *Parainfluenza virus type 2* | *Coxsackievirus-B6* |  |
| **DNA viruses(25 types)** | | |
| *Human bocavirus type 1* | *Human adenovirus B7 type* | *Herpesvirus type 1* |
| *Human bocavirus type 2* | *Human adenovirus B55 type* | *Herpesvirus type 2* |
| *Human bocavirus type 3* | *Human adenovirus Group C* | *Varicella-zoster virus* |
| *Human bocavirus type 4* | *Human adenovirus C1 type* | *Epstein-Barr virus* |
| *Parvovirus B-19* | *Human adenovirus C2 type* | *Cytomegalovirus* |
| *Human adenovirus* | *Human adenovirus C5 type* | *Human herpesvirus 6/7 type* |
| *Human adenovirus Group B* | *Human adenovirus Group E* | *BK Polyomavirus* |
| *Human adenovirus B3 type* | *Human adenovirus E4 type* | *JC Polyomavirus* |
| *Human adenovirus Group D* |  |  |
| **Bacteria (60 species)** | | |
| *Streptococcus pneumoniae* | *Mycobacterium fortuitum* | *Burkholderia cepacia* |
| *Streptococcus pyogenes* | *Mycolicibacterium smegmatis* | *Burkholderia mallei* |
| *Streptococcus agalactiae* | *Mycobacterium marinum* | *Burkholderia pseudomallei* |
| *Staphylococcus aureus* | *Mycobacterium ulcerans* | *Stenotrophomonas maltophilia* |
| *Enterococcus faecium* | *Mycobacterium haemophilum* | *Acinetobacter baumannii* |
| *Enterococcus faecalis* | *Mycobacterium gordonae* | *Moraxella catarrhalis* |
| *Tropheryma whipplei* | *Mycobacterium xenopi* | *Elizabethkingia meningoseptica* |
| *Bacillus cereus* | *Mycobacterium vaccae* | *Haemophilus influenzae* |
| *Bacillus anthracis* | *Mycobacterium malmoense* | *Haemophilus parainfluenzae* |
| *Corynebacterium pseudotuberculosis* | *Nocardia asteroides* | *Haemophilus haemolyticus* |
| *Corynebacterium diphtheriae* | *Nocardia brasiliensis* | *Pasteurella multocida* |
| *Trueperella pyogenes* | *Nocardia concava* | *Francisella tularensis* |
| *Rhodococcus hoagii* | *Nocardia gelsenkirchenensis* | *Bordetella pertussis* |
| *Mycobacterium tuberculosis complex* | *Neisseria meningitidis* | *Bordetella parapertussis* |
| *non-tuberculous mycobacteria* | *Escherichia coli* | *Bordetella avium* |
| *Mycobacterium avium* | *Salmonella enteritidis* | *Bordetella holmesii* |
| *Mycobacterium intracellulare* | *Klebsiella pneumoniae* | *Legionella pneumophila* |
| *Mycobacterium kansasii* | *Klebsiella oxytoca* | *Bacteroides fragilis* |
| *Mycobacteroides chelonae* | *Enterobacter cloacae* | *Proteus mirabilis* |
| *Mycobacterium abscessus* | *Serratia marcescens* | *Pseudomonas aeruginosa* |
| **Fungi (14 species)** | | |
| *Cryptococcus neoformans* | *Histoplasma capsulatum* | *Scedosporium apiospermum* |
| *Aspergillus fumigatus* | *Talaromyces marneffei* | *Schizophyllum commune* |
| *Pneumocystis jirovecii* | *Rhizomucor pusillus* | *Rhizopus oryzae* |
| *Candida albicans* | *Lichtheimia corymbifera* | *Trichosporon asahii* |
| *Cryptococcus gattii* | *Rhizopus microsporus* |  |
| **Others (11 species)** | | |
| *Mycoplasma pneumoniae* | *Ureaplasma parvum* | *Rickettsia rickettsii* |
| *Chlamydia pneumoniae* | *Ureaplasma urealyticum* | *Rickettsia tsutsugamushi* |
| *Chlamydia trachomatis* | *Rickettsia typhi* | *Rickettsia burneti* |
| *Chlamydia psittaci* | *Rickettsia prowazekii* |  |
| **Resistance genes/genotypes(379 genotypes)** | | |
| *KPC (67 genotypes)* | *VIM (71 genotypes)* | *VanC2* |
| *SME (5 genotypes)* | *GIM (2 genotypes)* | *VanD* |
| *IMI (19genotypes)* | *SPM* | *VanG* |
| *NMC* | *OXA-48 (40 genotypes)* | *VanE* |
| *GES (46 genotypes)* | *VanA* | *mecA* |
| *NDM (37 genotypes)* | *VanB* |  |
| *IMP (82 genotypes)* | *VanC1* |  |
